# A survey of current and past Pediatric Infectious Diseases fellows regarding training

**DOI:** 10.1186/1472-6920-11-72

**Published:** 2011-09-26

**Authors:** Miltiadis Douvoyiannis, Nathan Litman, Peter F Belamarich, David L Goldman

**Affiliations:** 1 Department of Pediatrics, Division of Infectious Diseases, Children's Hospital at Montefiore, 3415 Bainbridge Ave, Bronx, NY 10467, USA; 2 Department of Pediatrics, Children's Hospital at Montefiore, 3415 Bainbridge Ave, Bronx, NY 10467, USA

**Keywords:** fellowship, training, infectious diseases, pediatrics, education

## Abstract

**Background:**

The objectives of this study were to characterize the satisfaction of Pediatric Infectious Diseases fellows with their training and to understand how opinions about training have changed over time.

**Methods:**

Anonymous survey studies were conducted with questions designed to include areas related to the 6 ACGME core competencies. Surveys for current fellows were distributed by fellowship directors, while surveys for graduates were mailed to all individuals with Pediatric Infectious Diseases certification.

**Results:**

Response rates for current fellows and graduates were 50% and 52%, respectively. Most fellows (98%) and graduates (92%) perceived their overall training favorably. Training in most clinical care areas was rated favorably, however both groups perceived relative deficiencies in several areas. Current fellows rated their training in other competency areas (e.g., systems-based practice, research, and ethics) more favorably when compared to past graduates. Recent graduates perceived their training more favorably in many of these areas compared to past graduates.

**Conclusions:**

Pediatric Infectious Diseases fellowship training is well regarded by the majority of current and past trainees. Views of current fellows reflect improved satisfaction with training in a variety of competency areas. Persistent deficiencies in clinical training likely reflect active barriers to education. Additional study is warranted to validate perceived deficiencies and to establish consensus on the importance of these areas to infectious diseases training.

## Background

The American Board of Pediatrics established Pediatric Infectious Diseases (PID) board certification in 1994. In the United States, there are approximately 1000 PID board certified specialists [1]and 59 PID training programs with 151 PID fellows in training for the year 2007 (http://www.ama-assn.org last accessed in March 2009) [1]. To be board eligible, applicants must complete a 3 -year fellowship in an accredited program in the United States or Canada and meet the requirements for scholarly activity. Eligible applicants must pass a certifying examination.

Since its recognition as a board-certified subspecialty, there have been important changes to the practice of pediatric infectious diseases resulting in part from new technologies. Advances in medical therapy have resulted significant increases in the number of immunocompromised children (e.g. transplant recipients and patients with malignancies) at risk for infection. New diagnostics and therapeutics have enhanced our ability to evaluate and treat infectious diseases. At the same time, the internet has greatly enhanced access to the medical literature among practitioners. These changes in PID practice also are likely to have affected training of fellows.

The training of pediatric infectious diseases fellows has been also been directly affected by actions of the Accreditation Council for Graduate Medical Education and the American Board of Pediatrics. This includes the implementation in 1999 of minimum program requirements. These requirements were designed to help standardize medical training and detail 6 core competencies, including: patient care, medical knowledge, practice based-learning, interpersonal and communication skills, professionalism and systems-based practice.

Despite these changes in both practice and training, the satisfaction of Pediatric Infectious Diseases specialists with their fellowship training has not been studied. Two studies of primarily adult medicine infectious diseases practitioners were conducted prior to 2000 [2,3]. One of these studies [2] included some pediatric infectious diseases practitioners and identified a variety of training deficiencies. The purposes of this study then were to characterize the satisfaction of current PID fellows with their training and to understand how this assessment has changed over time. To this end, we surveyed and compared the satisfaction of current fellows and graduations with their training. We also compared the assessments of recent (2000-2007) and past (pre-2000) graduates

## Methods

### Survey

We devised surveys to assess the satisfaction of current fellows and graduates with their fellowship training (see additional file 1). Surveys included questions related to the six core competencies as outlined by the ACGME including: patient care, medical knowledge, practice-based learning, interpersonal skills, professionalism and systems-based practice. Questions also covered areas of deficiencies previously identified in adult infectious diseases and pediatric residency training programs [2-5]. The graduate survey was similar to the fellow's survey, but also contained additional questions related to research training and didactic courses (see additional file 2).

To evaluate satisfaction in areas of training, a 6-point Likert scale was used, where 1 = not at all, 2 = inadequate/not enough, 3 = inadequate/inappropriate experience, 4 = adequate, 5 = very well, 6 = too much time. To help the respondent recognize the meaning of a score of 6, it was set-off from the other scores by a line in the survey table (see additional files 1 and 2). To describe the information-seeking behaviors of trainees in addressing clinical problems, a 5-point Likert scale was used, where 1 = none, 2 = some, 3 = half, 4 = most and 5 = all of the time. Open-ended questions were also included to provide perspectives on duration and improvement of PID fellowship training. Both surveys were collected in anonymous fashion.

### Subjects and Recruitment

Surveys for current fellows were mailed in November 2008 and a reminder sent 5 weeks later to the 59 PID fellowship programs directors, identified on the Pediatric Infectious Diseases Society web site http://www.pids.org. Fellowship directors were asked to distribute the survey to their fellows. A second reminder was sent by e-mail as a web-based questionnaire http://www.surveymonkey.com to the fellows in June 2009. In conjunction with the American Board of Pediatrics, one thousand and thirty five graduates were identified as individuals with certification in Pediatric Infectious Diseases. Surveys were sent to 1005 individuals who had an address in the United States in April 2009 with a reminder sent 4-6 weeks later. In addition, a web-based format of the survey http://www.surveymonkey.com was sent to all 1035 certified specialists in August 2009.

### Analysis and Statistics

The percentage of respondents who considered their training adequate or better (a score 4 or more) and less than adequate (a score 1-3) were calculated. For the purpose of this analysis, we included scores of 6 (too much time) since training in theses areas was considered at least adequate. Areas in which more than 20% of respondents rated their training as less than adequate were considered deficient. Responses between current fellows and all graduates were compared. For comparisons related to HIV and transplantation, we restricted our graduate cohort to specialists who graduated after 1985 and 2000, respectively. Graduate responses were further analyzed by year of fellowship completion. For categorical data, a Fisher's exact test, odds ratio and corresponding 95% confidence intervals were calculated. P values < 0.05 were considered significant. All values were calculated using Graph Pad Software, Prism 5 (La Jolla, CA). The survey was approved by the Pediatric Research Committee at the Children's Hospital at Montefiore and exempted for review by the Institutional Review Board of Montefiore Medical Center and the Children's Hospital at Montefiore.

## Results

### Characteristics of Respondents

Of 151 fellows in training, seventy-six (50.3%) fellows responded. Eighteen out of 76 (24%) were 1^st^, 27 (36%) 2^nd ^and 28 (37%) 3^rd ^year fellows, while 3 did not indicate year of fellowship. Forty-six out of 76 (60%) planed an academic career, 21 (28%) were undecided, one intended to work in private practice, 2 respondents specified "other plans" and 6 did not indicate any plans. Of the 1035 PID board certified graduates with known mail or e-mail addresses, we received 536 (52%) responses. The majority of graduates (66%, 351/531) were employed by an academic institution, while 11% (60/531) were employed by the government and 10% (54/531) were in a private practice group.

### Satisfaction

Overall training was considered adequate (score of 4) by 41 (54%) or very good (score of 5) by 34 (45%) of 76 of current fellows. Of those graduates who responded to this question (n = 502), 236 (47%) considered overall training adequate (score of 4) while 227 (45%) considered it very good (score 5). A greater percentage of graduates considered their overall training less than adequate when compared with current fellows (8 vs. 1%, p = 0.048).

### Clinical Training

**Figure 1:**
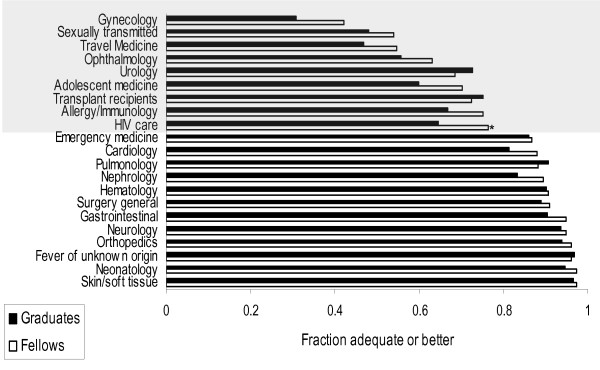
**Satisfaction of fellows and graduates with their training in a variety of clinical areas**. The proportion of respondents (fellows and graduates) who felt their training in a specific area was adequate is shown. Area shaded in gray represents areas in which 20% or more of fellows felt their training was inadequate. * P < 0.05 for comparison between fellows and graduates.

Current fellows considered their training in most aspects of patient care as adequate or better (score ≥ 4) (Figure 1). Training was felt to be excessive (score = 6) for orthopedic and transplant- associated infections by 9% (7/76) and 11% (8/76) of fellows, respectively. There was close agreement between graduates and current fellows with regard to their assessment of clinical training (Figure 1). Other than for HIV, there were no significant differences between the proportion of fellows and graduates, who perceived their training as adequate or better. More than 20% of fellows and graduates perceived their training as less than adequate (score 1-3) in the following areas: gynecology, sexually transmitted disease, travel medicine, ophthalmology, adolescent medicine, urology, transplantation, allergy and immunology and HIV care. The 3 areas most commonly rated 6 (too much training) were infections related to transplant medicine, orthopedics and skin/soft tissue by 10.5, 9.2 and 6.6% of fellows respectively. In all other areas excessive training was noted by 3% or less of respondents.

### Systems-based Practice

**Figure 2:**
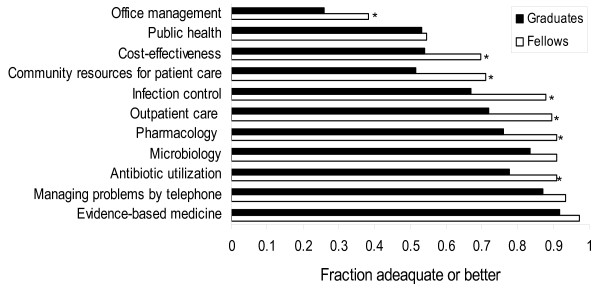
**Satisfaction of fellows and graduates with training in areas related to system-based practice**. The proportion of respondents (fellows and graduates) who felt their training in a specific area was adequate (score ≥ 4) is shown * P < 0.05 for comparison between fellows and graduates.

With regard to training in systems-based practice, at least 80% of fellows considered their training as adequate or better for many of the queried areas (Figure 2). More than 20% of current fellows and graduates indicated that their training was less than adequate in office management, public health, cost effectiveness and community resources for patients. Additional training areas that were identified by more than 20% of graduates as inadequate are shown (Figure 2). More than 85% of both fellows and graduates felt that their training in managing problems by phone was adequate. Approximately 17% (13/76) of current fellows compared with 4% of graduates considered their training in managing problems by phone as excessive (p < 0.001).

### Academic Training and Professionalism

**Figure 3:**
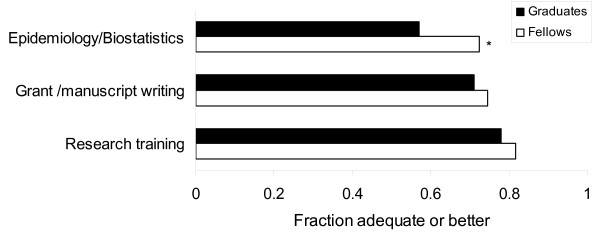
**Satisfaction of fellows and graduates with training in areas related to research**. * The proportion of respondents (fellows and graduates) who felt their training in a specific area was adequate (score ≥ 4) is shown. P < 0.05 for comparison between fellows and graduates.

**Figure 4:**
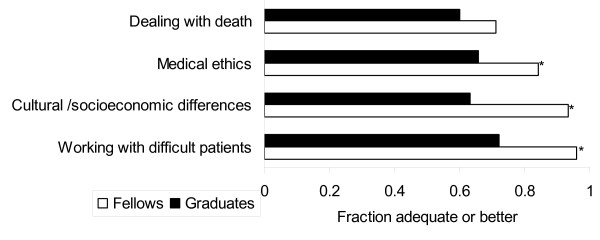
**Satisfaction of fellows and graduates with training in areas related to ethics, humanism and professionalism**. The proportion of respondents (fellows and graduates) who felt their training in a specific area was adequate (score ≥ 4) is shown * P < 0.05 for comparison between fellows and graduates.

Seventy percent or more of fellows and graduates considered their training in research and grant/manuscript writing as adequate or better (Figure 3). A greater proportion of fellows compared with graduates considered their training in epidemiology and biostatistics adequate or better. A greater proportion of fellows versus graduates felt training in these areas were adequate or better in medical ethics, dealing with cultural/socioeconomic differences and working with difficult patients (Figure 4).

Ninety-seven percent (74/76) of fellows were satisfied with their training in evidence-based medicine. However, half or less of the time (score 1-3) medical journals, guidelines or internet sources were used by 31%, 26% and 14% of fellows, respectively for clinical questions. Eighty-two percent of all fellows and 74% of graduates reported consulting a PID attending for clinical questions more than half of the time (score ≥ 4). Compared with fellows, graduates were more likely to report using a textbook as a reference more than 50% of the time (p = 0.04) and less likely to use the internet (p = 0.02).

### Fellowship Duration

**Figure 5:**
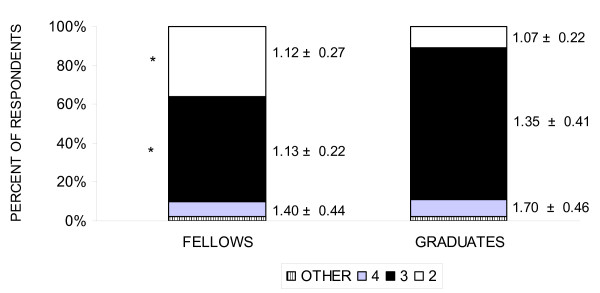
**Recommended duration for fellowship training**. The percent of fellows and graduates who recommended 2, 3, or 4 years of fellowship are shown. Numbers next to these stacked columns represents respective suggested average duration of clinical training (in years). * p < 0.01 for comparison between percent of fellows and graduates.

Thirty-six percent of fellows compared with 11% of graduates suggested 2 years as an ideal duration for fellowship training (p < 0.001) (Figure 5). Likewise a greater percentage of graduates (78%) compared with fellows (54%) suggested an ideal duration of 3 years for fellowship training. Of fellows who suggested 2, 3 or 4 years of fellowship training, academic career plans were reported by 17/27 (63%), 28/38 (74%) and 3/5 (60%), respectively.

### Suggestions for Improvement

The most frequent suggestions by fellows for improvement of the fellowship were more structured didactic lectures or review courses and more exposure to specific clinical areas. Other suggestions included more protected research time, more microbiology lab and more opportunities for international experience. We received 388 comments from graduates in response to the question "what one thing would you do to enhance your fellowship experience?" Most suggested increased training or experience in one of the following areas (in decreasing frequency): statistics/epidemiology, grant/manuscript writing, clinical laboratory/microbiology, international health experience, infection control, research training, sexually transmitted diseases, HIV, immunology, public health, travel medicine, adolescent medicine and parasitology. Other suggestions included (in decreasing frequency): better guidance on research/career, a better mentor, improved lecture series, more protected research time and enhanced research funding.

### Recent versus Past Graduates

We also characterized differences among responses of graduates trained before 2000 (past) and those trained from 2000 to 2007 (recent). Of the 536 graduates, 152 (28%) were recent graduates, 371 (69%) were past graduates and 13 (2%) did not specify their year of graduation. Greater percentages of past graduates spent the majority of their time in administration or were self-employed when compared with recent graduates (Table 1). Twenty-eight percent (42/152) of recent graduates versus 12% percent (45/354) of past graduates had additional degrees (PhD, MPH, MS) (p < 0.0001).

**Table 1:** Titles and employment characteristics of graduates, according to the period of fellowship completion

	All graduates (n = 536)	2000-2007 (n = 152)	Before 2000 (n = 371)	OR (95% CI)	*P*
Title	n = 532,				
MD	503 (95)	140 (92)	354 (95)		
DO	7 (1)	3 (2)	4 (1)		
MBBS	22 (4)	9 (6)	13 (4)		
Additional title	89 (17)	42 (28)	45 (12)	2.8 (1.7-4.4)	< 0.0001
PhD	29 (5)	15 (10)	14 (4)	2.8 (1.3-5.9)	0.01
MPH	47 (9)	25 (16)	22 (6)	3.0 (1.7-5.6)	0.0004
MS	16 (3)	5 (3)	10 (3)		
Primary employer	n = 531	n = 152	n = 369		
Academic institution	351 (66)	108 (71)	239 (65)		
Government/Federal	60 (11)	19 (12)	40 (11)		
Private practice group	54 (10)	17 (11)	34 (9)		
Private hospital (non-teaching)	17 (3)	5 (3)	12 (3)		
Pharmaceutical industry	14 (3)	1 (1)	12 (3)		
Self-employed	21 (4)	1 (1)	19 (5)	.012 (.02-.92)	0.01
Other	14 (3)	1 (1)	13 (4)		
Majority of time spent	n = 500	n = 144	n = 346		
Direct patient care	228 (46)	65 (45)	156 (45)		
Teaching/Research	173 (34)	62 (43)	109 (32)	1.6 (1.1-2.4)	0.01
Administration	59 (12)	4 (3)	55 (16)	0.2 (0.06-0.4)	< 0.0001
Other	40 (8)	13 (9)	26 (7)		

Data are No. (%), unless otherwise specified. Numbers may not add up since not all graduates answered all questions. Odds ratios and *p *values for statistically significant differences.

Recent graduates compared with past graduates more commonly rated their training as adequate or better in a variety of clinical and non-clinical training areas (Table 2). As part of the graduate survey, we also included questions related to specific courses taken during fellowship training. Fourteen and 32% of recent graduates reported taking the IDSA infection control course and a STD course, respectively compared with 5 and 22% of past graduates (p < 0.01 for both comparisons). Over 80% of both recent and past graduates reported taking courses in microbiology and over 25% reported taking courses in parasitology.

**Table 2:** Differences in satisfaction (score ≥ 4) among Pediatric Infectious Diseases specialists who completed their training between 2000-2007 versus those who completed their training before 2000

Areas of training	2000-2007	Before 2000	OR (95% CI)	*p*
Dealing with infectious diseases in				
Emergency medicine	137/149 (92%)	280/353 (79%)	2.9 (1.6-5.7)	0.0004
Hematology	142/151 (94%)	314/357 (88%)	2.1 (1.0-4.6)	0.04
Neurology	145/151 (96%)	316/357 (89%)	3.0 (1.2-7.1)	0.01
Transplantation	122/151 (81%)	222/352 (63%)	2.5 (1.6-3.9)	< 0.0001
Urology	119/149 (80%)	247/355 (70%)	1.7 (1.1-2.7)	0.02
Epidemiology/Biostatistics	98/151 (65%)	172/355 (48%)	2.0 (1.3-2.9)	0.0009
Public health	97/151 (64%)	192/357 (54%)	1.5 (1.0-2.3)	0.03
Outpatient care (clinic)	132/152 (87%)	266/357 (75%)	2.3 (1.3-3.8)	0.002
Managing problems by telephone	141/152 (93%)	245/358 (68%)	5.9 (3.1-11.3)	< 0.0001
Antibiotic utilization/control	131/152 (86%)	266/359 (74%)	2.2 (1.3-3.7)	0.002
Grant/manuscript writing	116/151 (77%)	230/359 (64%)	1.9 (1.2-2.9)	0.005
Practicing Evidence-Based medicine	143/152 (94%)	233/355 (66%)	8.3 (4.1-16.9)	< 0.0001
Community resources for patient care	96/149 (64%)	165/357 (45%)	2.1 (1.4-3.1)	0.0002
Working with difficult patients/families	124/152 (82%)	242/357 (68%)	2.1 (1.3-3.4)	0.001
Medical ethics	115/152 (76%)	220/357 (62%)	1.9 (1.2-3.0)	0.002
Cost-effectiveness	99/152 (65%)	177/358 (49%)	1.9 (1.3-2.8)	0.001
Office management	51/150 (34%)	80/358 (22%)	1.8 (1.2-2.7)	0.008

Shown are the fraction of individuals from each cohort who rated their training as adequate or better.

## Discussion

Results from this study indicate that current fellows perceive their training in a variety of core-competency areas more favorably than graduates. Differences between these cohorts were most prominent for competences outside of clinical care (e.g. ethics, systems-based practice, research). We suggest that these findings represent an improvement in training areas over time. An alternative explanation would be that graduates realize deficiencies in their training in the context of practice. However, the finding that a greater percent of recent graduates compared with past graduates rate their training as adequate or better in several non-clinical areas provides strong support for the notion that training has improved over time. The basis for these improvements in training remained to be formally demonstrated. Nonetheless, these improvements appear to be temporally associated with institution of ACGME core competencies and correlate to areas of emphasis by the ACGME. Thus, it seems reasonable to hypothesize that they are connected, but additional study is warranted.

Our results also indicate that current fellows and graduates found their training to be adequate or better in most areas of clinical care. The extent of agreement between fellows and graduates was quite remarkable with the exception of HIV and gynecology. Both fellows and graduates identified several areas of clinical care training as inadequate, including: gynecology, sexually transmitted disease, travel medicine, ophthalmology, adolescent medicine, urology, transplantation, allergy and immunology and HIV care. Organ transplantation and travel medicine have previously been identified as deficient by adult infectious diseases fellows/practitioners [2,3]. Some of these areas were previously noted as deficient in earlier studies of infectious disease practitioners. Our studies show an increase in the percent of recent compared with past fellows who have taken courses in Infection Control, a previously defined area of deficiency [2,3]. These findings suggest that programs are actively trying to address perceived deficiencies. Nonetheless, there appears to be persistent deficiencies that require active interventions to remediate. We suggest that additional objective assessment regarding the adequacy of training in these areas should be considered. Furthermore, assessment by the Pediatric Infectious Diseases community and the American Board of Pediatrics as to the relative importance of these elements to the practice of Pediatric Infectious Diseases may be warranted.

Telephone consultation has been demonstrated to be a significant component of PID practice [6]. However, a previous study suggested that only a minority of phone calls to a PID fellow were of educational value [7]. Among fellows, excessive training (score 6) was most commonly reported in managing problems by phone This likely reflects the amount of time fellows spent on the phone with antibiotic approvals, consults, etc. The perceived additional phone call management did not appear to negatively affect the overall satisfaction of fellows with their training, but additional study may be warranted.

Currently the duration of the PID fellowship training in the United States is 3 years. More than a third of fellows suggested a 2-year fellowship duration, while only 11% of graduates felt training should be 2 yrs. Among fellows, there were no apparent differences in plans for an academic career based on suggested duration of fellowship training. In a recent survey, 42% of all pediatric subspecialists indicated they would have chosen a 2-year fellowship without research if they had the option [8].

The limitations of this survey included the relatively small number of the fellow respondents, though we achieved a higher response rate than anticipated compared with previous studies of this type [2,3]. It should be noted that the responses to our surveys represent perceived deficiencies and do not necessarily reflect adequacy of training. Other limitations include the short time (5-6 months) that some of the 1^st ^year fellows had been trained at the time of the survey. Finally, the precise contribution of perspective change (versus changes in educational experience) to the observed differences among fellows and graduates are difficult to define.

## Conclusions

We conclude that the PID fellowship training is highly regarded by the current fellows and graduates. Our findings further suggest an improvement in training, especially in non-clinical areas over time, which may be related to the institution of ACGME core competencies. Specific training deficiencies appear to persist in fellowship training and may identify areas of needed improvement. We are hopeful that the results from this work can provide useful input for further improvements to Pediatric Infectious Diseases training.

## Abbreviations

PID: Pediatric Infectious Diseases; IDSA: Infectious Diseases Society of America; ACGME: Accreditation Council for Graduate Medical Education; HIV: Human Immunodeficiency Virus.

## Financial disclosures

Miltiadis Douvoyiannis reports no financial disclosures.

Nathan Litman reports no financial disclosures.

Peter F. Belamarich reports no financial disclosures.

David L. Goldman reports no financial disclosures.

## Conflicts of interest

Miltiadis Douvoyiannis reports no conflicts of interest.

Nathan Litman reports no conflicts of interest.

Peter F. Belamarich reports no conflicts of interest.

David L. Goldman reports no conflicts of interest

## Authors' contributions

MD helped design, send out, and analyze survey results. He also co-drafted the manuscript. NL helped design and analyze survey results. He also edited manuscript. PFB helped design and analyze survey results. He also edited manuscript. DLG supervised entire project. He helped design and analyze survey results. He also co-drafted the manuscript. All authors read and approved the final manuscript

## Pre-publication history

The pre-publication history for this paper can be accessed here:

http://www.biomedcentral.com/1472-6920/11/72/prepub

## Supplementary Material

Additional file 1 **Fellows survey**. Survey sent to current Pediatric Infectious Diseases fellows. Click here for file

Additional file 2 **Survey for graduates**. Survey sent to graduates of a Pediatric infectious Diseases training program. Click here for file
