# Supplementary material for: A survey of current and past Pediatric Infectious Diseases fellows regarding training
Source: BMC Med Educ. 2011 Sep 26;11:72. doi: 10.1186/1472-6920-11-72 (PMC3188472; doi:10.1186/1472-6920-11-72)
Supplement: Additional file 1 — Fellows survey. Survey sent to current Pediatric Infectious Diseases fellows. [file 1472-6920-11-72-S1.PDF]

Pediatric Infectious Diseases Fellowship – Questionnaire

Please indicate:

Year of Fellowship: ☐ First ☐ Second ☐ Third

Plans after Fellowship: ☐ Academic career ☐ Private practice

☐ Other : ☐ To be decided

A. Please rank your training in the following areas, during your Fellowship in Pediatric Infectious Diseases.

(1 = not at all, 2 = inadequate, not enough training, 3 = inadequate, inappropriate experience **4 = adequate, 5 = very well,** 6 = too much time)

1.	Dealing with infectious diseases in						
	Adolescent medicine	1	2	3	**4**	**5**	6
	Allergy/Immunology	1	2	3	**4**	**5**	6
	Cardiology	1	2	3	**4**	**5**	6
	Emergency medicine	1	2	3	**4**	**5**	6
	Fever of unknown origin	1	2	3	**4**	**5**	6
	Gastrointestinal	1	2	3	**4**	**5**	6
	Gynecology	1	2	3	**4**	**5**	6
	HIV care	1	2	3	**4**	**5**	6
	Hematology	1	2	3	**4**	**5**	6
	Neonatology	1	2	3	**4**	**5**	6
	Nephrology	1	2	3	**4**	**5**	6
	Neurology	1	2	3	**4**	**5**	6
	Oncology						
	Ophthalmology	1	2	3	**4**	**5**	6
	Orthopedics	1	2	3	**4**	**5**	6
	Pulmonology	1	2	3	**4**	**5**	6
	Sexually transmitted diseases	1	2	3	**4**	**5**	6
	Surgery general	1	2	3	**4**	**5**	6
	Skin/soft tissue	1	2	3	**4**	**5**	6
	Transplant recipients	1	2	3	**4**	**5**	6
	Urology	1	2	3	**4**	**5**	6
2.	Epidemiology/ Biostatistics	1	2	3	**4**	**5**	6
3.	Microbiology laboratory techniques	1	2	3	**4**	**5**	6
4.	Pharmacology of antimicrobials	1	2	3	**4**	**5**	6
5.	Public health	1	2	3	**4**	**5**	6
6.	Infection control	1	2	3	**4**	**5**	6
7.	Outpatient care (clinic)	1	2	3	**4**	**5**	6
8.	Travel medicine	1	2	3	**4**	**5**	6
9.	Managing problems by telephone	1	2	3	**4**	**5**	6
10.	Antibiotic utilization/control	1	2	3	**4**	**5**	6
11.	Research training	1	2	3	**4**	**5**	6
12.	Grant / manuscript writing	1	2	3	**4**	**5**	6
13.	Practicing Evidence-Based Medicine	1	2	3	**4**	**5**	6
14.	Community resources for patient care	1	2	3	**4**	**5**	6
15.	Cultural/ socioeconomic differences	1	2	3	**4**	**5**	6
16.	Working with difficult patients/families	1	2	3	**4**	**5**	6
17.	Dealing with death	1	2	3	**4**	**5**	6
18.	Medical ethics	1	2	3	**4**	**5**	6
19.	Cost-effectiveness	1	2	3	**4**	**5**	6
20.	Office management	1	2	3	**4**	**5**	6
**21.**	**Overall training**	1	2	3	**4**	**5**	

**B.** What sources of information you use during the Fellowship to answer your clinical questions, **(except medication dosage)**	1 =none of the time	2=some of the time	3 = half of the time	4 =most of the time	5 = all of the time
a) Attending (Peds ID)	1	2	3	4	5
b) Fellow (Peds ID)	1	2	3	4	5
c) Pharmacist	1	2	3	4	5
d) Journal articles	1	2	3	4	5
e) Practice guidelines	1	2	3	4	5
f) Textbook	1	2	3	4	5
g) Internet source: i.e Uptodate, Pub Med, Cochraine,	1	2	3	4	5
h) PDA	1	2	3	4	5
i) Other: i.e Hariet Lane Nelson's handbook	1	2	3	4	5

C. How many years a Pediatric Infectious Diseases Fellowship should last?

☐ 2 years (How much time for clinical training?)
(How much time for research?)

☐ 3 years (How much time for clinical training?)
(How much time for research?)

☐ 4 years (How much time for clinical training?)
(How much time for research?)

D. Finally, what one thing would you do to enhance your Fellowship experience?

E. Additional comments?

Thank you very much for your participation
