# Supplementary material for: A survey of current and past Pediatric Infectious Diseases fellows regarding training
Source: BMC Med Educ. 2011 Sep 26;11:72. doi: 10.1186/1472-6920-11-72 (PMC3188472; doi:10.1186/1472-6920-11-72)
Supplement: Additional file 2 — Survey for graduates. Survey sent to graduates of a Pediatric infectious Diseases training program. [file 1472-6920-11-72-S2.PDF]

Pediatric Infectious Diseases Fellowship Graduates – Questionnaire

The Children's Hospital at Montefiore

Please indicate one or more:	☐ MD ☐ DO ☐ MBBS<br>☐ PhD ☐ MS ☐ MPH
Year of Fellowship completion:	☐
Country of Fellowship training:	☐ USA ☐ Canada ☐ Other country
Primary Employer:	☐ Academic institution<br>☐ Government / Federal<br>☐ Private group practice<br>☐ Private hospital (non-teaching)<br>☐ Pharmaceutical industry<br>☐ Self-employed
Majority of time spent in:	☐ Direct Patient care<br>☐ Teaching / Research<br>☐ Administration<br>☐ Other

A. Please rank your satisfaction with your fellowship training in the following areas.
(1 = not at all, 2 = inadequate, not enough training, 3 = inadequate, inappropriate experience
4 = adequate, 5 = very well, 6 = too much time)

1.	Dealing with infectious diseases in						
	Adolescent medicine	1	2	3	**4**	**5**	6
	Allergy/Immunology	1	2	3	**4**	**5**	6
	Cardiology	1	2	3	**4**	**5**	6
	Emergency medicine	1	2	3	**4**	**5**	6
	Fever of unknown origin	1	2	3	**4**	**5**	6
	Gastrointestinal	1	2	3	**4**	**5**	6
	Gynecology	1	2	3	**4**	**5**	6
	HIV care	1	2	3	**4**	**5**	6
	Hematology	1	2	3	**4**	**5**	6
	Neonatology	1	2	3	**4**	**5**	6
	Nephrology	1	2	3	**4**	**5**	6
	Neurology	1	2	3	**4**	**5**	6
	Oncology	1	2	3	**4**	**5**	6
	Ophthalmology	1	2	3	**4**	**5**	6
	Orthopedics	1	2	3	**4**	**5**	6
	Pulmonology	1	2	3	**4**	**5**	6
	Sexually transmitted diseases	1	2	3	**4**	**5**	6
	Surgery general	1	2	3	**4**	**5**	6
	Skin/soft tissue	1	2	3	**4**	**5**	6
	Transplant recipients	1	2	3	**4**	**5**	6
	Urology	1	2	3	**4**	**5**	6
2.	Epidemiology/ Biostatistics	1	2	3	**4**	**5**	6
3.	Microbiology laboratory techniques	1	2	3	**4**	**5**	6
4.	Pharmacology of antimicrobials	1	2	3	**4**	**5**	6
5.	Public health	1	2	3	**4**	**5**	6
6.	Infection control	1	2	3	**4**	**5**	6
7.	Outpatient care (clinical)	1	2	3	**4**	**5**	6
8.	Travel medicine	1	2	3	**4**	**5**	6
9.	Managing problems by telephone	1	2	3	**4**	**5**	6
10.	Antibiotic utilization/control	1	2	3	**4**	**5**	6
11.	Research training	1	2	3	**4**	**5**	6
12.	Grant / manuscript writing	1	2	3	**4**	**5**	6
13.	Practicing Evidence-Based Medicine	1	2	3	**4**	**5**	6
14.	Community resources for patient care	1	2	3	**4**	**5**	6
15.	Cultural/ socioeconomic differences	1	2	3	**4**	**5**	6
16.	Working with difficult patients/families	1	2	3	**4**	**5**	6
17.	Dealing with death	1	2	3	**4**	**5**	6
18.	Medical ethics	1	2	3	**4**	**5**	6
19.	Cost-effectiveness	1	2	3	**4**	**5**	6
20.	Office management (administrative)	1	2	3	**4**	**5**	6
**21.**	**Overall satisfaction**	1	2	3	**4**	**5**	

(1 = not at all, 2 = inadequate, not enough training, 3 = inadequate, inappropriate experience
4 = adequate, 5 = very well, 6 = too much time)

B. Please indicate

i) Did your Fellowship include a course or rotation in

Parasitology	□ Yes	□ No
STDs	□ Yes	□ No
Microbiology	□ Yes	□ No

ii) What of the following could have improved your reaserch training:

more protected time	□ Yes	□ No
a different mentor	□ Yes	□ No
a formal course	□ Yes	□ No
more-secure funding	□ Yes	□ No

iii) Have you ever taken the infection control course on the IDSA website? □ Yes □ No

iv) Overall the pediatric infectious diseses Fellow- Attending interactions were

□ 1 = poor □ 2 = fair □ 3 = good □ 4 = very good □ 5 = excellent

C. What sources of information you used during the Fellowship to answer your clinical questions, **(except medication dosage)**

	1 =none of the time	2=some of the time	3 = half of the time	4 =most of the time	5 = all of the time
a) Attending (Peds ID)	1	2	3	4	5
b) Fellow (Peds ID)	1	2	3	4	5
c) Pharmacist	1	2	3	4	5
d) Journal articles	1	2	3	4	5
e) Practice guidelines	1	2	3	4	5
f) Textbook	1	2	3	4	5
g) Internet source: i.e Uptodate, Pub Med, Cochrane,	1	2	3	4	5
h) PDA	1	2	3	4	5
i) Other (i.e Hariet Lane, Nelson Handbook	1	2	3	4	5

D. How many years a Pediatric Infectious Diseases Fellowship should last?

☐ 2 years (How much time for clinical training?)
(How much time for research?)

☐ 3 years (How much time for clinical training?)
(How much time for research?)

☐ 4 years (How much time for clinical training?)
(How much time for research?)

E. Finally, what one thing would you do to enhance your fellowhip experience?

F. Additional comments?

Thank you very much for your participation
